# The Neutrophil Function in Severe Sepsis/Septic Shock Patients With Mods

**DOI:** 10.1186/2197-425X-3-S1-A519

**Published:** 2015-10-01

**Authors:** N Klinngam, C Sittipunt, S Poonyathawon, P Chatrkaw, S Tachaboon, N Srisawat

**Affiliations:** Department of Anesthesiology and Critical Care, Faculty of Medicine, Chulalongkorn University, Bangkok, Thailand; Division of Pulmonary and Critical Care, Department of Medicine, Chulalongkorn University, Bangkok, Thailand; Division of Nephrology, Department of Medicine, Chulalongkorn University, Bangkok, Thailand

## Introduction

It is clearly shown that neutrophil play a key role in the innate immune response to infection in the animal model. However, little is known about the neutrophil function during severe sepsis in the real clinical practice.

## Objectives

To compare the neutrophil function which comprise of neutrophil chemotaxis activity and myeloperoxidase enzyme (MPO) activity between healthy subjects and critically ill patients with severe sepsis/septic shock.

## Methods

Severe sepsis/septic patients who were admitted into ICU at King Chulalongkorn Memorial Hospital within 48 hours after the onset of sepsis were recruited. We divided the patients into 4 groups (gr.) based on type of organ failure, 10 patients for each: without organ failure gr., respiratory failure gr. (ARDS), kidney failure gr. (AKI), and multiple organ dysfunction gr. (MODS). We collected the blood sample at the enrollment time. We measured the chemotaxis function by measurement the percentage of neutrophil migration, and tested MPO activity by using Quantikine human MPO immunoassay (ELISA). We used the standard definition criteria to define severe sepsis/septic shock status. We defined survival status based on alive or not at hospital discharge time.

## Results

Patients who presented with severe sepsis/septic shock and healthy volunteers were enrolled. Severe sepsis/septic shock group revealed significantly decreased of percentage neutrophil migration compared to healthy volunteers group (42 ± 20 % vs 85 ± 4%, P < 0.001). However when stratified by type of organ failure, there was no significant difference of chemotaxis among type of organ failure, P = 0.48. There was a trend that in survivor group would have better chemotaxis function but still no reach statistical significance (48 ± 20% vs 34 ± 17%, P = 0.48).The MPO activity in severe sepsis/septic shock group was significantly higher than healthy group (3,207 ± 1,796 ng/ml vs 100 ± 17 ng/ml, P = 0.003). Again, there was no significant difference of MPO activity among type of organ failure, P = 0.50.

## Conclusions

This study was the first time which could demonstrate the immunosuppressive effect of severe sepsis/septic shock patients by decreasing chemotaxis function. This finding should contribute to find out the novel treatment for improvement of neutrophil function and the survival rate in the future.
